# Phenotypic variability in cases with CACNA1A mutation

**DOI:** 10.1007/s00431-025-06062-3

**Published:** 2025-03-20

**Authors:** Sema Bozkaya-Yilmaz, Nihal Olgac-Dundar, Nargiz Aliyeva, Atilla Ersen, Pinar Gencpinar, Mesut Gungor, Ayse Semra Hiz, Uluc Yis, Gamze Sarikaya-Uzan, Esra Sarigecili, Serkan Kirik, Ilknur Erol, Seyda Besen, Hulya Kayilioglu, Senay Haspolat, Osman Kipoglu, Arzu Ekici, Sevim Turay, Ayse Tosun, Muge Ayanoglu, Aysegul Danis, Fatma Hancı, Yasar Bekir Kutbay, Berk Ozyilmaz, Bulent Kara

**Affiliations:** 1 Department of Pediatric Neurology, Bursa State Hospital, Bursa, Turkey; 2 Department of Pediatric Neurology, Faculty of Medicine, Katip Celebi University, Izmir, Turkey; 3 Department of Pediatric Neurology, Liv Bonadea Hospital, Baku, Azerbaijan; 4 Department of Pediatric Neurology, Private Clinic, Izmir, Turkey; 5 Department of Pediatric Neurology, Faculty of Medicine, Selcuk University, Konya, Turkey; 6 Department of Pediatric Neurology, Faculty of Medicine, Dokuz Eylul University, Izmir, Turkey; 7 Department of Pediatric Neurology, Adana State Hospital, Adana, Turkey; 8 Department of Pediatric Neurology, Faculty of Medicine, Firat University, Elazig, Turkey; 9 Department of Pediatric Neurology, Faculty of Medicine, Baskent University, Adana, Turkey; 10 Department of Pediatric Neurology, Faculty of Medicine, Mugla Sitki Kocman University, Mugla, Turkey; 11 Department of Pediatric Neurology, Faculty of Medicine, Akdeniz University, Antalya, Turkey; 12 Department of Pediatric Neurology, Mehmet Akif Training and Research Hospital, Sanliurfa, Turkey; 13 Department of Pediatric Neurology, Private Clinic, Bursa, Turkey; 14 Department of Pediatric Neurology, Faculty of Medicine, Duzce University, Duzce, Turkey; 15 Department of Pediatric Neurology, Faculty of Medicine, Adnan Menderes University, Aydin, Turkey; 16 Department of Pediatric Neurology, Faculty of Medicine, Abant Izzet Baysal University, Bolu, Turkey; 17 Genetic Diseases Diagnosis Center, Izmır State Hospital, Izmir, Turkey; 18 Department of Pediatric Neurology, Faculty of Medicine, Kocaeli University, Kocaeli, Turkey

**Keywords:** *CACNA1A*, Genetic, Epilepsy, Neurodevelopmental disorder

## Abstract

The purpose of this study was to enhance understanding of *CACNA1A* gene variants by elucidating the clinical profiles of patients with different variants. The overlapping features and varying phenotypic characteristics of these neurological disorders pose challenges for clinicians. A data collection form was utilized to gather clinical features, examination details, and treatment information associated with *CACNA1A* variants. Thirty-one patients were included in the study from 11 different clinics in Turkey. Cases were assessed by comparing their information with existing literature. The study initially included 32 patients from 29 families, with 31 patients meeting the inclusion criteria. Clinical manifestations ranged from congenital onset hypotonia to motor seizures. Within the group of patients, 87% were diagnosed with epilepsy, 61% had neurodevelopmental defects, 32% experienced ataxia, 22% had eye movement problems, 16% suffered from migraines, and 13% had recurrent encephalopathy. Thirty percent of individuals exhibited cerebellar atrophy. A subset of individuals exhibited various forms of cognitive impairment and different kinds of ataxia.

*Conclusion*:* CACNA1A* variants can lead to structural and functional abnormalities in the Cav2.1 channels, resulting in paroxysmal and/or chronic clinical presentations. The overlapping phenotypes and variable features among family members suggest the influence of environmental factors and modifier genes. A thorough understanding of the range of phenotypic variants and the difficulties encountered by medical professionals is essential for precise diagnosis and efficient treatment approaches in various neurological conditions. Additional research is necessary to clarify the underlying mechanisms that contribute to the various presentations of these variants.

**Table:** 

**What is known:** *• Variants in the CACNA1A gene disrupt calcium signaling, thereby impacting fundamental developmental processes such as neuronal differentiation, migration, and synapse formation.* *• Variants in the CACNA1A can lead to neurodevelopmental disorders characterized by intellectual disability, learning difficulties, memory challenges, and problems in social interaction.* **What is new:** *• Instances of intrafamilial variability in CACNA1A variants have been identified, with differing clinical manifestations exhibited by affected family members.* *• Incomplete penetrance is a phenomenon that may occur, as neurodevelopmental or neuropsychiatric findings are not exhibited by some patients with CACNA1A variants.*

**What is known:**

*• Variants in the CACNA1A gene disrupt calcium signaling, thereby impacting fundamental developmental processes such as neuronal differentiation, migration, and synapse formation.*

*• Variants in the CACNA1A can lead to neurodevelopmental disorders characterized by intellectual disability, learning difficulties, memory challenges, and problems in social interaction.*

**What is new:**

*• Instances of intrafamilial variability in CACNA1A variants have been identified, with differing clinical manifestations exhibited by affected family members.*

*• Incomplete penetrance is a phenomenon that may occur, as neurodevelopmental or neuropsychiatric findings are not exhibited by some patients with CACNA1A variants.*

## Introduction

The *CACNA1A* gene is responsible for encoding the alpha-1A subunit of the P/Q-type voltage-gated calcium channel, which plays a crucial role in regulating calcium influx primarily in the central nervous system. Located on chromosome 19 and consisting of 47 exons, the *CACNA1A* gene encodes the Cav2.1 protein, a crucial component of the Cav2.1 channel. This channel comprises four subunits, including the alpha-1 subunit, which features four homologous domains (1–4) composed of six transmembrane segments (S1–S6). Notably, the S4 segment acts as the voltage sensor, whereas the S5–S6 segments contribute to the ion-conducting pore [1].

*CACNA1A* gene variants have been associated with a diverse array of neurological disorders, highlighting the critical role of this gene in normal neuronal function. Episodic ataxia, characterized by recurrent episodes of imbalance and uncoordinated movements, has been linked to *CACNA1A* variants, with distinct subtypes exhibiting different clinical presentations [2]. Hemiplegic migraine, a severe form of migraine with additional neurological symptoms like paralysis, is also associated with *CACNA1A* variants [3]. Additionally, spinocerebellar ataxia, an inherited progressive disorder affecting coordination and movement, and early infantile epileptic encephalopathy, a severe epilepsy disorder that begins in infancy, have been attributed to *CACNA1A* gene variants [4]. Understanding the diverse phenotypes associated with *CACNA1A* variants is crucial for accurate diagnosis and tailored treatment approaches. Although there is no cure for these disorders, management strategies aim to alleviate symptoms and improve quality of life. Treatment options may include preventive medications to reduce the frequency and severity of episodes, anti-seizure medications for seizure control, and supportive therapies such as physical and occupational therapy to manage motor impairments.

Gaining a comprehensive understanding of the *CACNA1A* gene and its role in neurological disorders is crucial for unraveling underlying mechanisms. By studying the structure and function of the calcium channel and its impacts on *CACNA1A*, researchers and clinicians can develop improved diagnostic tools and treatment strategies. Genetic testing can identify specific variants, guide treatment decisions, and predict disease progression. Furthermore, understanding the pathophysiology of *CACNA1A*-related disorders may lead to the identification of potential drug targets and more effective treatments. We aimed to contribute to the existing literature with this study, broadening knowledge about *CACNA1A* and its implications in neurological disorders.

## Material and method

In order to deepen our understanding of *CACNA1A*-related disorders and their clinical implications, a data collection form was created to capture relevant clinical features, examination details, and treatment information associated with *CACNA1A* variants. To facilitate this multi-center study, a call for participation was announced in pediatric neurology clinics across Turkey. This data was then analyzed by comparing it with the existing literature, contributing to a comprehensive understanding of *CACNA1A*-related disorders.

Most of the (Patients #18-#28) Multi-gene NGS panels analysis was carried out at Tepecik Hospital, Genetic Diseases Evaluation Center. A Custom Target Capture NGS Panel named “Large Epilepsy NGS Panel” (Celemics, Inc., Seoul, Korea) was used for molecular genetic evaluation (https://intronsaglik.com.tr/wp-content/uploads/2022/01/celemics.pdf). This NGS Panel covered all coding regions and exon–intron boundaries of the included genes. For this purpose, genomic DNA samples were extracted from 2 mL peripheral blood samples. A target capture-based NGS panel kit was used for library preparation. The Target Capture NGS study was performed on an Illumina MiSeq NGS System (Illumina, Inc., San Diego, CA, USA) using MiSeq Reagent Nano Kit v3 (Illumina, Inc., San Diego, CA, USA). FASTQ sequencing files were collected and imported into “SEQ” variant analysis software (Genomize, Istanbul, Turkey). Data were analyzed using “SEQ” variant analysis software (Genomize, Istanbul, Turkey) with the GRCh37 (h19) reference genome. Coverage data was obtained from the “CDS Coverage % Table” of the SEQ variant analysis software, and the read depth of each variant was checked from the SEQ variant analysis software as well as IGV. Variants for which all ClinVAR submissions were Benign or Likely Benign were excluded. Interpretation of variants was performed according to standards and guidelines published by the American College of Medical Genetics (ACMG).

The data collection form included demographic variables, initial and subsequent clinical findings (ataxia, seizure, epilepsy, migraine, hypotonia, autism, intellectual disability, ocular findings), laboratory results (MRI, EEG, and genetic results), and treatment information.

Thirty-one patients were included in the study from 11 different pediatric neurology clinics across Turkey. The inclusion criteria for the study specified that patients must have genetic results indicating pathogenic or likely pathogenic variants, or variants of uncertain significance (VUS) that were included based on clinical overlap. Considering the previously discussed intrafamilial variability, patients who carried the same variants as their asymptomatic or mildly symptomatic parents, as well as those with VUS variants, were not excluded from the study. However, patients with benign or likely benign variants were excluded from the study.

Written informed consent was obtained from the parent or legal guardian of each patient and the study was conducted in accordance with the ethical principles outlined in the Declaration of Helsinki. Ethics committee approval was also obtained from Health Sciences University Tepecik Training and Research Hospital (Date: 02/20/22).

## Results

A total of 32 patients from 29 families, including 2 sets of siblings (patients #12, #13, and #14; and patients #28 and #29), initially participated in this study, representing 11 different centers. After screening for eligibility, 31 patients were deemed suitable for inclusion. One patient with a likely benign variant was excluded due to the presence of the same variant in the asymptomatic mother and weak clinical evidence linking it to the *CACNA1A* gene. The father of patients #12, #13, and #14 also exhibited epileptic and hemiparetic attacks. Similarly, the mother of patients #28 and 29, who carried the same variant, experienced frequent recurring bouts of dizziness. However, these individuals were not included in the pediatric age study group.

The age range of our patient cohort at the time of examination and diagnosis varied from 4 to 204 months. The earliest observed manifestation was congenital onset hypotonia, while the latest manifestation presented as a motor seizure at 144 months. Table 1 provides details regarding the ages of our patients at examination and diagnosis, age of initial findings, presence of epilepsy and/or febrile seizures, EEG findings (generalized or focal), neurodevelopmental problems, ataxia, migraine, other episodic events (such as recurrent encephalopathies, nystagmus, and paroxysmal tonic upgaze), and the presence of cerebellar atrophy on brain MRI. Table 2 displays information on the variants, diagnostic methods, clinical significance, novelty, genetic studies on parents, specific treatments, and treatment responses.

**Table 1:** Clinical and MRI-EEG findings of patients

Pt no	Age (y)	Age of diagnosis (y)	Age of first neurological event (m)	Epilepsy/FS	Seizure type	EEG	Neuro-developmental delay	Ataxia	Migraine	Brain MRI	Findings of Brain MRI	Additional neurological findings	Ophthalmological findings
1	7	5	9	Yes/ +	Focal, GTC	Generalized	MID	CA	-	Normal	-	-	-
2	2	2	0	Yes/ +	Focal	Focal	GDD, hypotonia	-	-	Abnormal	EOCA	Hemiplegia	Nystagmus, PTU
3	2	1	13	Yes	Myoclonic	Generalized	MID, hypotonia	-	-	Normal		-	-
4	2	0.3	4	Yes	Atonic, GTC	Generalized	GDD	-	-	Abnormal	EOCA	-	Nystagmus, PTU
5	17	17	24	Yes	Tonic	Generalized	MID	CA	-	Abnormal	EOCA	Recurrent encephalopathy	-
6	10	9	6	Yes	Atonic, Focal	Focal	MID, ASD, hypotonia	CA, EA2	-	Normal	-	Recurrent encephalopathy	PTU
7	5	5	60	Yes	Absence, GTC	Generalized	-	-	-	Normal	-	-	-
8	2	1	8	Yes	Absence	Focal	-	-	-	Normal	-	-	-
9	3	1	5	Yes	Tonic	Focal	GDD, hypotonia	-	-	Abnormal	EOCA*	Strabismus	-
10	3	1	5	Yes	Infantile spasm	Generalized	GDD, hypotonia	-	-	Normal	-	-	-
11	3	1	10	Yes	Focal	Normal	-	-	-	Normal	-	-	-
12	21	14	144	Yes	Focal	Focal	MID	Late-onset ataxia	FHM	Abnormal	EOCA	-	-
13	12	8	144	No	-	-	-	-	FHM	Abnormal	EOCA	Numbness in hands and feet	-
14	17	10	96	Yes	GTC	Generalized	-	EA2	-	Abnormal	EOCA	Recurrent encephalopathy	-
15	6	5	54	Yes	Atonic	Generalized	-	EA2	-	Normal	-	-	-
16	6	5	5	Yes/ +	GTC	Generalized	-	-	-	Normal	-	-	-
17	1	1	12	No	Atonic	Normal	-	-	-	Normal	-	-	-
18	2	1	10	No/ +	GTC	Focal	-	-	-	Normal	-	-	-
19	11	10	12	Yes	Atonic	Generalized	MID	EA2	Migraine	Normal	-	-	-
20	6	6	36	Yes	GTC	Focal	-	-	-	Normal	-	-	PTU
21	5	5	48	Yes	Absence	Generalized	MID	-	-	Normal	-	-	-
22	16	7	84	Yes	GTC	Normal	-	-	-	Normal	-	-	-
23	14	4	48	Yes	GTC	Focal	MID, ASD	-	-	Normal	-	-	-
24	8	5	8	Yes	GTC, SE	Focal	GDD	CA, tremor	Migraine	Normal	-	-	Nystagmus, PTU
25	3	2	24	Yes	Atonic, Focal	Generalized	GDD	CA, tremor	-	Normal	-	-	PTU
26	6	5	1	Yes	GTC	Focal	GDD, hypotonia	-	-	Abnormal	-	Microcephaly	-
27	14	14	72	Yes	-	Normal	-	-	HM?	Normal	-	Recurrent encephalopathy	-
28	7	7	18	Yes	GTC	Normal	MID	-	-	Normal	-	-	-
29	4	4	12	No/ +	GTC	Normal	MID	-	-	Normal	-	Cataract	-
30	1	0.9	1	Yes	Absence, tonic	Generalized	GDD, hypotonia	CA	-	-	-	-	-
31	12	8	4	Yes	GTC	Generalized	GDD, hypotonia	-	-	Abnormal	EOCA	-	Nystagmus, PTU

*Pt* patient, *no* number, *FS* febrile seizure, *GTC* generalized tonic clonic seizure, *SE* status epilepticus, *EEG* electroencephalography, *MID* mild intellectual disability, *GDD* global developmental delay, *ASD* autistic spectrum disorder, *CA* congenital ataxia, *EA2* episodic ataxia type 2, *FHM* familial hemiplegic migraine, *HM* hemiplegic migraine, *EOCA* early onset cerebellar atrophy, *PTU* paroxysmal tonic upgaze disorder

**Table 2:** Genetic results and treatment outcomes

Pt	Diagnostic method	Variant	Protein change	HGVS	Exon	Novel variant	Pathogenicity of the variant	Zygosity	Mother	Father	Specific agent(s) used	Treatment response	
							Clinvar/ACMG				For treatment	Acetazolamide	Verapamil
1	MGP	c.4687G > A	p.(Val1563Met)	NM_001127222.2:c.4687G > A (p.Val1563Met)	29	No	VUS/VUS	Heterozygote	No mut	Same mut	Acetazolamide	Good response	-
2	WES	c.4046G > A	p.(Arg1349Gln)	NM_001127222.2:c.4046G > A (p.Arg1349Gln)	25	No	Pathogenic/VUS	Heterozygote	No mut	No mut	Acetazolamide, verapamil	Good response	Partial response
3	MGP	c.6409G > C	p.(Asp2137His)	NM_001127222.2:c.6409G > C (p.Asp2137His)	45	No	VUS/VUS	Heterozygote	No mut	Same mut	Acetazolamide	Good response	-
4	MGP	c.3058G > A	p.(Glu1020Lys)	NM_001127222.2:c.3058G > A (p.Glu1020Lys)	19	No	-/Pathogenic	Heterozygote	NA	NA	-	-	-
5	WES	c.1862A > T	p.Ser621Phe	NM_001127222.2:c.1862 T > C (p.Ser621Phe)	14	Yes	VUS/VUS	Heterozygote	No mut	No mut	Acetazolamide	Unknown*	-
6	WES	c.7261_7262del	p.(Pro2421Val)	NM_001127222.2:c.7261_7262del (p.Pro2421Glyfs*81)	47	No	-/Likely Pathogenic	Heterozygote	No mut	Same mut	Acetazolamide	No response	-
7	NA	c.6748A > C	p.(Ser2250Arg)	NM_001127222.2:c.6748A > C (p.Ser2250Arg)	46	No	-/VUS-Likely Pathogenic	Heterozygote	NA	NA	-	-	-
8	NA	c.6733 T > G	p.(Trp2245Gly)	NM_001127222.2:c.6733 T > G (p.Trp2245Gly)	46	No	-/VUS	Heterozygote	No mut	No mut	-	-	-
9	WES	c.2947G > A	G983S	NM_001127222.2:c.2947G > A (p.Gly982Ser)	19	Yes	VUS/Pathogenic	Heterozygote	Same mut	No mut	-	-	-
10	WES	c.7424G > A	Q2775A	NM_001127222.2:c.7424G > A (p.Gly2475Asp)	47	Yes	-/VUS	Heterozygote	No mut	No mut	-	-	-
11	WES-MGP	c.1781A > C	Lys594Thr	NM_001127222.2:c.1781A > C (p.Lys594Thr)	13	No	VUS/VUS	Heterozygote	NA	NA	-	-	-
12	WES	c.653C > T	Ser218Leu	NM_001127222.2:c.653C > T (p.Ser218Leu)	5	No	Pathogenic/Pathogenic	Homozygote	No mut	Same mut	Acetazolamide	No response	-
13	WES	c.653C > T	Ser218Leu	NM_001127222.2:c.653C > T (p.Ser218Leu)	5	No	Pathogenic/Pathogenic	Homozygote	No mut	Same mut	-	-	-
14	WES	c.653C > T	Ser218Leu	NM_001127222.2:c.653C > T (p.Ser218Leu)	5	No	Pathogenic/Pathogenic	Homozygote	No mut	Same mut	-	-	-
15	WES	c.5083_5094del		NM_001127222.2:c.5083_5094del (p.Cys1695_Ile1698del)	33	No	-/VUS	Heterozygote	NA	NA	Acetazolamide	Good response	-
16	MGP	c.6400C > T	R2134C	NM_001127222.2:c.6400C > T (p.Arg2134Cys)	45	No	Conflicting/VUS	Heterozygote	NA	NA	-	-	-
17	MGP	c.3547G > A	V1183I	NM_001127222.2:c.3547G > A (p.Val1183Ile)	20	No	VUS/VUS	Heterozygote	NA	NA	-	-	-
18	MGP	c.6595C > T	R2199W	NM_001127222.2:c.6595C > T (p.Arg2199Trp)	46	No	VUS/VUS	Heterozygote	No mut	Same mut	-	-	-
19	MGP	c.5626C > T	Arg1876Trp	NM_001127222.2:c.5626C > T (p.Arg1876Trp)	38	No	VUS/VUS	Heterozygote	No mut	Same mut	-	-	-
20	MGP	c.3634G > T	E801G	NM_001127222.2:c.3634G > T (p.Asp1212Tyr)	21	Yes	VUS/VUS	Heterozygote	Same mut	No mut	-	-	-
21	MGP	c.6743C > T	E667K	NM_001127222.2:c.6743C > T (p.Ser2248Leu)	46	No	VUS/VUS	Heterozygote	Same mut	No mut	-	-	-
22	MGP	c.2950C > G	R984G	NM_001127222.2:c.2950C > G (p.Arg984Gly)	19	Yes	VUS/VUS	Heterozygote	Same mut	No mut	-	-	-
23	MGP	c.5854G > A	Val1952Met	NM_001127222.2: c.5854G > A (p.Val1952Met)	40	No	VUS- Likely Pathogenic	Heterozygote	NA	NA	-	-	-
24	MGP	c.4174G > A	V1392M	NM_001127222.2:c.4174G > A (p.Val1392Met)	26	No	VUS/Likely Pathogenic	Heterozygote	NA	NA	Acetazolamide	Partial response	-
25	MGP	c.1999G > A	E667K	NM_001127222.2:c.1999G > A (p.Glu667Lys)	16	No	VUS/Pathogenic	Heterozygote	Same mut	No mut		-	-
26	MGP	c.3547G > A	V1183I	NM_001127222.2:c.3547G > A (p.Val1183Ile)	20	No	VUS/VUS	Heterozygote	NA	NA		-	-
27	WES	c.6406G > C	Asp2136His	NM_001127222.2:c.6406G > C (p.Asp2136His)	45	No	VUS/VUS	Heterozygote	NA	NA	Acetazolamide	Good response	-
28	WES	c.4033C > T	Arg1345Ter	NM_001127222.2:c.4033C > T (p.Arg1345*)	25	No	Pathogenic/Pathogenic	Heterozygote	Same mut	No mut	-	-	-
29	WES	c.4033C > T	Arg1345Ter	NM_001127222.2:c.4033C > T (p.Arg1345*)	25	No	Pathogenic/Pathogenic	Heterozygote	Same mut	No mut	-	-	-
30	MGP	c.3857 T > G	Val1282Gly	NM_001127222.2:c.3857 T > G ( p.Val1286Gly)	23	No	-/Likely Pathogenic	Heterozygote	NA	NA	Acetazolamide	Partial response	-
31	MGP	c.4186G > A	Val1396Met	NM_001127222.2:c.4186G > A (p.Val1395Met)	26	No	-/Pathogenic	Heterozygote	No mut	No mut	Acetazolamide	Good response	**-**

*MGP* multi-gene panel for epilepsy, *WES* whole exome sequencing, *VUS* variant of uncertain significance, *mut* mutation, *NA* not available

In our patient cohort, epilepsy was observed in 87% of cases (*n* = 27), neurodevelopmental anomalies in 61% (*n* = 19), ataxia in 32% (*n* = 10), eye movement disorders (such as nystagmus and PTU) in 22% (*n* = 7), migraine in 16% (*n* = 5), and recurrent encephalopathy in 13% (*n* = 4). Cerebellar atrophy was detected in 25% of patients (*n* = 8). Among patients with neurodevelopmental anomalies, 22% (*n* = 7) exhibited moderate cognitive impairment, while 19% (*n* = 6) presented with global developmental disorders and hypotonia, and 9% (*n* = 3) had global developmental disorders alone. Regarding ataxia, 9% (*n* = 3) of patients had episodic ataxia, while 16% (*n* = 5) had early-onset ataxia. Notably, two patients displayed tremors in conjunction with early-onset ataxia.

**Fig. 1:**
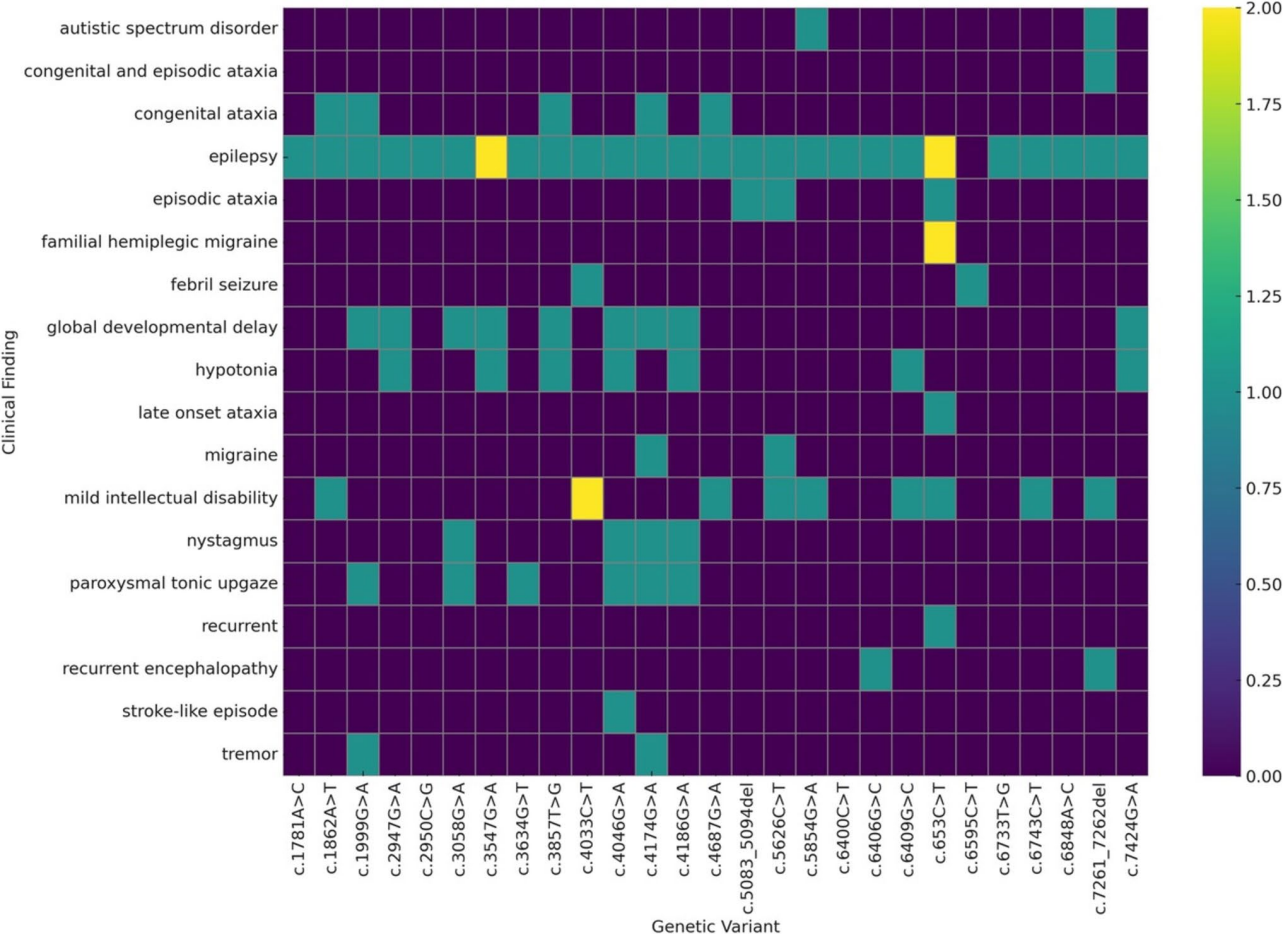
Common features of the patients

**Fig. 2:**
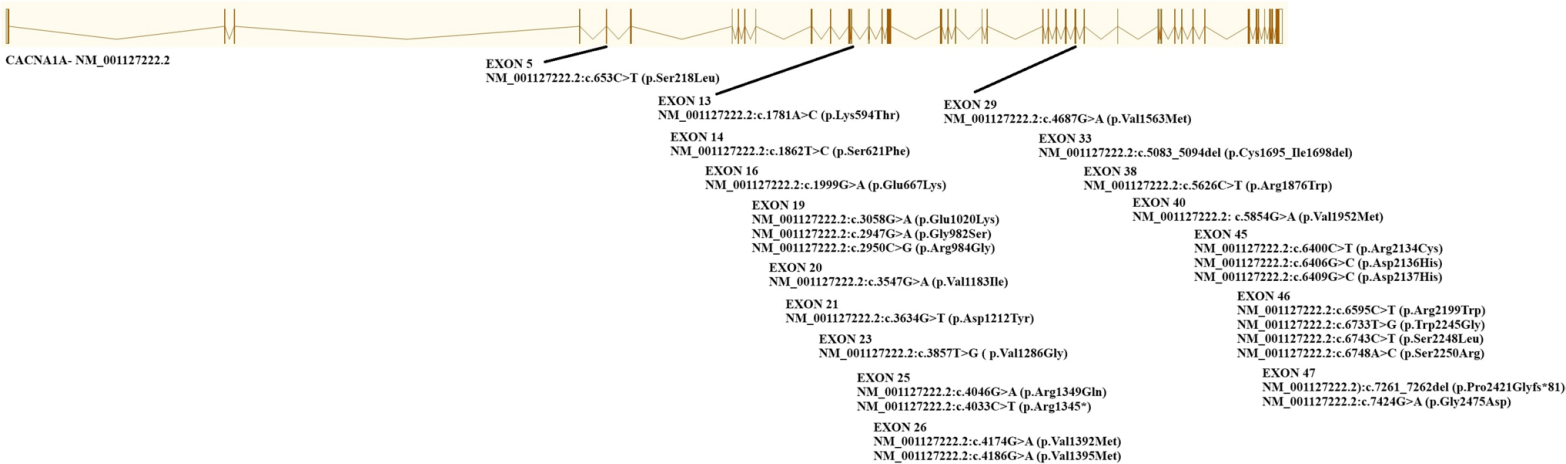
Illustration of variants

In Fig. 1, the common features of the patients are illustrated. Figure 2, on the other hand, presents a depiction of the variants.

## Discussion

The *CACNA1A* gene exhibits heterozygous point mutations that give rise to a wide range of episodic and/or permanent neurological manifestations. Triplet mutations in the same gene are responsible for a consistent presentation of late onset spinocerebellar ataxia type 6 [5]. Point mutations occur when a single nucleotide base is changed, altering the amino acid sequence of the Cav2.1 protein. Depending on the mutation’s location and nature, this can lead to a loss or gain of protein function. Some point mutations disrupt normal folding or stability of the protein leading to a loss of function while others alter the channel’s gating properties, resulting in gain of function that increases calcium influx and neurotransmitter release. Familial hemiplegic migraine (FHM) episodic ataxia (EA2) and some cases of spinocerebellar ataxia have been linked to point mutations in the *CACNA1A* gene. FHM has generally been linked to missence gain-of-function mutations while EA2 has been associated with loss-of-function mutations including small deletions and truncating mutations [6, 7].

Frameshift mutations occur when nucleotide bases are inserted or deleted from the DNA sequence, resulting a shift in the reading frame of the gene. This often produces a truncated or altered protein that may not function properly. In the case of *CACNA1A*, frameshift mutations can lead to a loss of function of the Cav2.1 channel, which can disrupt the neurotransmitter release and contributing to neurological symptoms. Frameshift mutations have been observed in some individuals with early-onset ataxia.

Although functional studies were not conducted in this study, our aim was to discuss the putative effects of the mutation on the structure and function of the Cav2.1 channel, based on in silico predictions or previous studies involving similar mutations. We acknowledge the limitation of our study in this regard, and further genetic tests would be required to provide a more comprehensive understanding. Our study included a higher number of epilepsy patients compared to previous research, which can be attributed to the ease of using the multi gene panel for epilepsy. Recent articles have reported developmental delay, ataxia, and hypotonia as the most prevalent phenotypes [4, 8].

More than 476 different Likely pathogenic (196), Pathogenic (280) variants in *CACNA1A* gene have been reported in the ClinVAR database. Despite various efforts, a clear genotype–phenotype association had not yet been established. In our patient group, among 31 patients, 17 had variant of uncertain significance (VUS). The inclusion of these variants in the study was based on the resemblance of their phenotypes to those previously reported in the scientific literature. The inclusion of these variants into our study on *CACNA1A* disease in the pediatric age group is necessary for a comprehensive understanding of this complex condition. These VUS variants represent genetic variations with uncertain implications, emphasizing the need for ongoing research. By incorporating these variants, we acknowledge the intricacy and heterogeneity of *CACNA1A* disease.

Additionally, we included patients in our study who exhibited detected variants in their parents but did not manifest clear symptoms associated with the *CACNA1A* variant. Segregation studies may not always yield conclusive information, as parents carrying *CACNA1A* variants could either be asymptomatic or display very mild and nonspecific features that do not meet the diagnostic criteria for any of the recognized classical *CACNA1A*-associated conditions [9].

All the phenotypes associated with *CACNA1A* have been addressed separately in our discussion, thoroughly examining the shared and distinctive characteristics observed in our presented cases in comparison to those previously documented in the literature.

### Epilepsy and developmental and epileptic encephalopathy type 42 (DEE 42)

Epilepsy is a complex neurological disorder with genetic factors implicated in approximately 65–80% of cases. Variants in the CACNA1A gene play a significant role in genetic epilepsy, as they impair the function of Cav2.1 channels. This can disrupt calcium signaling and neuronal excitability, potentially contributing to epilepsy development. However, the specific mechanisms by which CACNA1A variants lead to epilepsy are not fully understood and may differ based on the mutation and its impact on channel function.

*CACNA1A* gene variants have been associated with various seizure disorders, including recurrent febrile seizures, early-onset febrile and afebrile status epilepticus, generalized seizures (such as absence seizures, myoclonic-astatic seizures, and tonic–clonic seizures), focal seizures, and rarely epileptic spasms. According to Alehabib et al., the spectrum of seizures ranges from generalized tonic–clonic seizures being the most common, followed by absence seizures and myoclonic-astatic seizures [10]. Additionally, *CACNA1A* variants have been associated with early-infantile developmental and epileptic encephalopathy and migratory focal epilepsy. Jiang et al. discovered that some individuals with Lennox Gastaut syndrome had de-novo variants in the *CACNA1A* gene. These variants could either result in a gain or loss of function of the calcium channel [11].

In patients suspected of having epileptic encephalopathy, characterized by the presence of multiple seizure types and accompanying psychomotor retardation, as well as in those exhibiting cerebellar signs such as nystagmus, paroxysmal dystonia, or ataxia, or in individuals with a positive family history for hemiplegic migraine/ataxia, *CACNA1A* sequencing should be considered [10, 12–14].

In their review study, Alehabib et al. found that individuals within the same family who carry a variant in the *CACNA1A* gene may not necessarily develop the same type of seizures or they may not experience at all. The presence or absence of seizures can vary among affected family members who share the same pathogenic variant in the gene [10]. In our study, we observed generalized seizures in one of the siblings carrying the c.653C > T variant, while focal motor seizures were observed in the other sibling. The father carrying the same variant exhibited behavior disturbances and seizures. The intrafamilial variability associated with CACNA1A variants is noteworthy and has been documented in several studies. For instance, Angeli et al. reported a wide variability of clinical features among relatives carrying a CACNA1A missense pathogenic variant [15]. Carriers can present with a spectrum of conditions, ranging from severe epileptic encephalopathy to episodic ataxia, or cerebellar ataxia accompanied by intellectual deficiency. A striking observation was the total absence of symptoms in three relatives who carried the pathogenic variant, highlighting the concept of incomplete penetrance. Such incomplete penetrance has also been suggested in previous studies, including two families noted in the Epi4K Consortium [16]. Notably, a case involving maternal mosaicism was reported, where a CACNA1A pathogenic variant was inherited from an asymptomatic mother. Additionally, Damaj et al. described a family with apparent incomplete penetrance, involving episodic ataxia type 2 (EA2) and epilepsy, though clinical examinations were not conducted for asymptomatic relatives [17]. Incomplete penetrance and intrafamilial variability should be kept in mind in the context of CACNA1A to help prevent underdiagnosis.

Our study included 23 patients (74%) diagnosed with epilepsy. Among them, two of our patients had a history of recurrent febrile seizures, while one patient had a single febrile seizure. One of our patients had a single unprovoked atonic seizure, while another patient posed challenges in distinguishing between paroxysmal tonic upgaze and motor seizure (patient #6). Similar to findings in existing literature, our study included a patient with recurrent status epilepticus (patient #24), and patients with tonic, tonic–clonic, myoclonic, atonic seizures as well as one patient presenting with infantile spasms. Most of our patients with epilepsy were either in remission or under control with a single medication. Generally, medication regimens that align with those described in the literature were used (valproate, carbamazepine, oxcarbazepine, levetiracetam, acetazolamide, topiramate) Notably, our patient #24 with recurrent status epilepticus had improvement with cannabidiol (CBD) treatment. To our knowledge, this treatment modality has not been previously reported in the literature for *CACNA1A* cases. Additionally patient #26, who suffered from refractory focal seizures, benefited from treatment with pregabalin.

The use of pregabalin treatment has been discussed in calcium channel-related psychiatric disorders and multiple sclerosis; however, its application in epilepsy treatment has not been reported [18, 19]. Patient #31 with refractory epilepsy required vagus nerve stimulation (VNS) in addition to treatment with valproate, clonazepam, topiramate, and sultiam. The use of VNS in the management of refractory epilepsy in *CACNA1A* cases has been previously reported in the literature.

### Hemiplegic migraine, stroke-like episodes, and strokes

Hemiplegic migraine (HM) is a rare neurological disorder characterized by severe and recurrent headaches and temporary paralysis and weakness on one side of the body. When HM occurs without a clear association or family history, it is classified as spontaneous hemiplegic migraine (SHM). Familial hemiplegic migraine (FHM) is an inherited form linked to genetic variants, primarily in the *CACNA1A* gene [20]. FHM variants are typically missense mutations, resulting in a gain of function potentially alteration in calcium ion regulation and neuronal excitability.

Research shows individuals with HM can experience changes in consciousness and behavior, hemiparesis/hemiplegia, speech impairments, and ataxia triggered by factors such as emotional stress or minor head trauma. Neuroimaging studies have detected brain oedema during these episodes, along with slowing detected in electrophysiological examinations on the affected side [21].

Though symptoms may be temporary in some cases, rehabilitation needs persist throughout a person’s lifetime. Variants in the *CACNA1A* gene lead to abnormal neurotransmitter release or altered synaptic transmission, disrupting the delicate balance of neuronal activity necessary for proper brain function. Furthermore, *CACNA1A* variants may affect the metabolic demands of brain cells, potentially causing cellular damage or loss of function [22, 23]. In their ultrastructural study conducted on *CACNA1A* variant FHM1 patients, Dziewulska and Kierdaszuk observed microvascular changes, suggesting that the disease is not only functional but also a structural vascular disorder [24]. *CACNA1A* disorder should be considered part of the evaluation for pediatric strokes, particularly when the brain lesions do not align with typical vascular territories [25].

In our study, c.653C > T variant, which was previously associated with familial hemiplegic migraine and coma presentation with minor head trauma, was detected in three siblings (patients #12, #13, #14). Two siblings presented with hemiplegic migraine while the third sibling experienced recurrent encephalopathy attacks. The father, who carried the same variant as the three siblings had recurrent hemiparesis attacks and epilepsy.

Patient 2 was carrying c.4046G > A variant. This variant was listed as “Pathogenic” in the ClinVAR database. The patient was presented with global developmental delay, hypotonia, nystagmus, and paroxysmal tonic upgaze; a stroke-like episode occurred. No vascular pathology was detected, but a hemiplegic condition developed necessitating rehabilitation.

In patient #19, a heterozygous c.5626C > T variant was detected. This variant was not listed in the ClinVAR database, and it was evaluated as VUS according to the ACMG criteria (PM2, PP2, PP3). Patient #27 was carrying a heterozygous c.6406G > C variant. This variant was not listed in the ClinVAR database, and it was evaluated as VUS according to the ACMG criteria (PM2, PP2). The patient presented with headaches that did not meet the criteria for migraine diagnosis. These headache episodes were accompanied by symptoms such as vertigo and unsteady walking. He has also recurrent encephalopathy attacks. Another patient, Patient #6, was carrying a heterozygous C.7261_7262delCCInsGT variant, which was not listed in the ClinVAR database, and it was evaluated as VUS according to the ACMG criteria (PM2, PP2). The presentation did not align with migraine criteria. However, the patient experienced frequent recurrent episodes of vertigo and cognitive impairment.

The three patients (#21, #29, and #8) with heterozygous VUS variants were reported here since all of these variants had extremely low frequencies in population databases and computational prediction tools (silico predictors) supported a deleterious effect on the gene. Recently, it is recommended to record and annually re-analyze such rare and potentially pathogenic variants, which may be clinically relevant. Since these patients at least have clinical findings such as headaches and migraine symptoms without hemiplegic features or any auras, it is possible that these rare variants may appear pathogenic in the future.

### Neuropsychiatric/neurodevelopmental features

*CACNA1A* gene variants can affect neurodevelopment and contribute both neurodevelopmental and neuropsychiatric conditions. These variants disrupt calcium signaling thereby impacting crucial developmental processes such as neuronal differentiation, migration, and synapse formation. Individuals with *CACNA1A* mutations may display intellectual impairment (ID). *CACNA1A* gene mutations are more likely to cause faulty synaptic plasticity and atypical neurotransmitter release, namely involving glutamate and GABA. These mutations disrupt the normal communication between neurons. People with ID may experience challenges in learning, problem-solving, reasoning, and memory. Moreover, *CACNA1A* variants can cause delays in various aspects of neurodevelopment including motor skills, language acquisition, and social interaction.

In a family study involving 79 individuals with autism spectrum disorder (ASD), including 77 trios and 2 quartets, *CACNA1A* has also been demonstrated to exhibit ASD-causing properties [26]. While *CACNA1A* variants alone may not directly cause autistic spectrum disorders (ASD), they have been reported individuals with ASD alongside other genetic and environmental factors. The specific relationship between *CACNA1A* variants and ASD is still under investigation.

In their study involving 44 patients who had their *CACNA1A* variant genetically confirmed, Indelicato et al. found that seven of the ten patients who provided early developmental history exhibited psychomotor milestone delays [12]. Poor school performance was observed in eight out of 22 patients for whom school history was available.

Eight patients suffered from psychological disorders such as psychosis, anxiety, personality disorder, and attention deficient hyperactivity disorder (ADHD). Neurophysiological testing revealed cognitive deficits in 22 out of 23 patients.

In our study, among 31 patients, 11 did not exhibit any neurodevelopmental or neuropsychiatric findings. Due to participation of patients from 11 different centers across our country, objective neurodevelopmental testing could not be performed. However, evolutions by clinicians were taken into consideration. There were 8 patients with mild cognitive impairment among the group. In one patient, mild cognitive impairment accompanied hypotonia, while another patient exhibited mild cognitive impairment coexisted with symptoms of autism spectrum disorder. In yet another patient, mild cognitive impairment, hypotonia, and symptoms of autism spectrum disorder were observed. Additionally, in 11 patients, global developmental delay frequently accompanied hypotonia.

### Episodic ataxia type 2 (EA2) and other type ataxias

Episodic ataxia (EA) is a neurological condition characterized by recurring episodes of instability, poor coordination and affected walking pattern often accompanied dizziness, vertigo, and headaches. Episodic Ataxia Type 2 (EA2) is related to genetic changes in *CACNA1A* gene among others, such as *ATP1A2* gene. *CACNA1A* mutations affecting neuronal excitability manifest in EA2. *CACNA1A* mutations seen in EA2 disease impair calcium channel function, leading to disruption of neurotransmission and causing firing defects in Purkinje cells. which leads to abnormal signaling in the cerebellum and episodic attacks of ataxia [7].

In episodic ataxias, the variants typically result in gain-of-function changes after the channel has been opened, causing paroxysmal episodes of ataxia rather than continuous symptoms in congenital forms. *CACNA1A* variants that impair the function of the Cav2.1 calcium channel disrupt the normal release of neurotransmitters, particularly glutamate, thus affecting communication between neurons. As a result, there is a continuous decline in the cerebellum and the neuronic regions surrounding it leading to progressive aggravation of symptoms.

In nonprogressive early-onset ataxias caused by *CACNA1A* variants, there are often missence mutations that do not completely abolish the Cav2.1 channel function but instead result in altered channel properties. Usually, these modifications result in an enhanced sensitivity to voltage in the channel or a change in its gating dynamics, allowing certain neurons in the cerebellum to become excessively excitable.

This hyperexcitability can disrupt the normal processing of sensory information and coordination of movement but does not cause progressive degeneration [27, 28].

According to the findings of Martínez-Monseny et al.’s study, among the *CACNA1A-*related phenotypes, nonprogressive early-onset ataxia is correlated with developmental delay and dysmorphic features, thus presenting as a distinct syndromic neurodevelopmental disorder. In our study, we also observed developmental delay in all cases with early-onset ataxia [29].

Patient #5, who has a novel mutation (c.4687G > A variant) exhibited early-onset ataxia along with cerebellar atrophy. In another case (patient #24) carrying the likely pathogenic c.4714 variant previously associated with episodic ataxia, distinct features such as pronounced early-onset ataxia, head titubation, and tremor were observed.

Again, patient #25 carrying the pathogenic c.1999G > A missence variant previously associated with EA, it is observed the presence of early-onset ataxia. We recognized episodic ataxia in two patients (patients #16 and #19) and early-onset ataxia in a patient (patient #6) carrying variant previously reported as VUS. Our ataxic cases except for the patient with the novel mutation had no cerebellar atrophy up until the date of evaluation. The absence of detectable cerebellar atrophy may be attributed to it not reaching a noticeable extent on macroscopic neuroimaging methods. Or it could suggest that that the underlying mechanism of these ataxias may not involve significant structural changes in the cerebellum. Instead, it is possible that the dysfunction is primarily related to functional or biochemical abnormalities, rather than observable anatomical changes. Additional investigations into the etiology of these ataxias may contribute to the discovery of precise mechanisms involved.

### Other presentations

Benign paroxysmal torticollis (BPT), benign paroxysmal vertigo (BPV), and benign paroxysmal tonic upgaze (BTU) may be associated with *CACNA1A* variants. In a cross-sectional study conducted by Humbertclaude et al., eight families out of fifty patients with BPT, BPV, and BTU were found to have *CACNA1A* variants [30].

*CACNA1A* variants disrupt the normal functioning of the *CACNA1A* gene, leading to abnormal neuronal activity in specific brain regions responsible for controlling head and neck movements. This disruption results in sudden and recurrent episodes of involuntary head tilting or turning in BPT. Similarly, BPV is also linked to *CACNA1A* variants. The calcium channels in the vestibular system and/or inner ear which are responsible for balance and spatial orientation are affected in these variants. The dysfunctional calcium channels can cause brief and recurring periods of dizziness, spinning sensations, or unsteadiness.

In addition, it is possible that the *CACNA1A* gene variants might induce BTU, an uncommon illness characterized by frequent periods of upward gazing accompanied with abnormal eye movements like nystagmus. The definite mechanism by which *CACNA1A* variants lead to this condition is not thoroughly understood yet, but it is believed to comprise abnormal functioning of the brainstem and the oculomotor system.

Patient #31, who carried a pathogenic c.4186G > A variant, had BTU and BPT in her medical history. The patient has also suffered from ongoing nystagmus. Similarly, in patient #2 with c.4046G > A variant, which previously described as VUS in the literature, the patient exhibited PTU and nystagmus. We observed most of the patients who have nystagmus (3 of 4) had cerebellar atrophy.

Spinocerebellar ataxia type 6 (SCA6) is a type of progressive degeneration of the cerebellum. Ataxia and/or lack of coordination can be seen. Abnormal expansion of CAG repeats in the *CACNA1A* gene leads to SCA6. Although clinical findings suggestive of infantile presentation have been identified in SCA6, the long-term clinical course of our patients is unknown; therefore, we refrain from attributing symptoms to this classification and do not discuss them under the umbrella of SCA6.

Hemiconvulsion-hemiplegia-epilepsy syndrome is also associated with *CACNA1A* variants. We did not provide detailed descriptions as we do not have patients with these conditions in our study.

### Treatment

In *CACNA1A*-related disorders, although there may not have been a sufficient number of large-scale randomized controlled trials, there are medication options that have been demonstrated to be effective through case reports, case series, and observational studies. These studies have contributed to the evidence base and identified medications that show positive effects in managing symptoms.

According to Indelicato and Boesch’s intensive study of *CACNA1A*-related channelopathies, one feasible option is acetazolamide for the chronic or paroxysmal symptoms related to *CACNA1A* [31]. Acetazolamide works by blocking carbonic anhydrase, an enzyme that plays a crucial role in maintaining body bicarbonate and acid–base balance. By decreasing bicarbonate levels, acetazolamide reduces neuron excitability. On the other hand, acetazolamide has some side effects such as gastrointestinal complaints, drowsiness and fatigue, electrolyte imbalances, and nephrolithiasis.

We have patients who have benefitted from acetazolamide treatment, as well as those who experienced partial benefits. Patient #2, who has stroke, epilepsy, nystagmus, and PTU, as well as patient #12, who has ataxia among the three siblings, and patient #15 who has epilepsy and episodic ataxia, and patient #31 experiencing global developmental disorder and epilepsy, nystagmus, tonic upgaze, and torticollis, have all benefited from acetazolamide treatment. Furthermore, patient #27, carrying 6406G > C variant and suffering from recurrent encephalopathy attacks, has shown promising results with the acetazolamide treatment. On the other hand, patient #1, who has epilepsy and early-onset ataxia, patient #3, who has epilepsy and developmental disorder, patient #24, who is followed for early-onset ataxia, epilepsy, and migraines, and patient #30, who has early-onset ataxia and epilepsy, have all reported partial benefits from acetazolamide treatment.

Verapamil, a calcium channel blocker, has emerged as a potential treatment option for individuals with *CACNA1A* variants. These variants disrupt the normal functioning of calcium channels, leading to increased neuronal excitability and neurotransmitter release. By inhibiting the calcium channels, verapamil aims to modulate neuronal activity and potentially alleviate symptoms associated with *CACNA1A*-related disorders [32]. Numerous studies have demonstrated encouraging outcomes in using verapamil in managing specific symptoms, such as migraine, ataxia, and episodic syndromes associated with *CACNA1A* variants. Verapamil’s ability to dampen excessive neuronal excitability and attenuate the underlying pathophysiological mechanisms provides a rationale for its use in this context [33].

Patient #2, who presented with a stroke-like attack, achieved an improvement in consciousness after administering verapamil.

The literature highlights flunarizine and topiramate as potential prophylactic treatments for migraine and aminopyridines for EA2. Moreover, given the encouraging outcome reported by Martakis and colleagues that N-acetyl-leucine acts as a new potential treatment for CACNA1A-related disorders, we predict that this compound could be beneficial for CACNA1A-related episodic ataxia [34]. We do not have any patients on these treatments so we are not going to address them. Nevertheless, it is important to note that the application of triptans in hemiplegic migraine may cause a stroke due to vasoconstriction [19].

Future treatments for *CACNA1A* mutations hold significant promise, particularly through innovative gene repair strategies. Antisense oligonucleotides (ASOs) represent a compelling therapeutic option, as they can be designed to target and modify specific RNA sequences associated with mutations [35]. This approach has shown success in treating conditions such as Duchenne muscular dystrophy and spinal muscular atrophy, indicating its potential for *CACNA1A*-related disorders. In addition to ASOs, CRISPR-based techniques, including gene activation, base editing, and prime editing, offer further avenues for addressing the underlying genetic defects. CRISPR-gene activation can enhance the expression of the *CACNA1A* gene, while base editing and prime editing allow for precise corrections of mutations, which may restore the production of functional CaV2.1 channels [36].

Furthermore, the potential suppression of the *CACNA1A* internal ribosomal entry site (IRES) function presents a novel therapeutic strategy for addressing disorders like spinocerebellar ataxia type 6 (SCA6), wherein the misregulation of α1ACT, a transcription factor, negatively impacts neuronal health [37].

Given the established complexity of *CACNA1A*-related diseases, these advanced gene editing techniques could provide personalized treatment solutions tailored to the specific mutations affecting individual patients. Collectively, the integration of these innovative strategies into therapeutic pipelines could pave the way for effective interventions aimed at mitigating the debilitating effects of *CACNA1A* mutations.

## Conclusions

*CACNA1A* variants can result in structural and functional abnormalities in Cav2.1 channels and give rise to paroxysmal and/or chronic clinical presentations. These presentations often exhibit overlapping phenotypes, with phenotypic features varying among family members due to the influence of environmental factors and modifier genes. Interestingly, even in cases of variant of uncertain significance (VUS) clinically relevant characteristics can still be observed. Consequently, these genetic disorders present notable challenges to clinicians.

In children with early onset of developmental delay and developmental regression, accompanied by intellectual disability-autism spectrum disorder, in cases presenting with stroke or stroke-like clinical symptoms without clear vascular etiology, *CACNA1A* variant should be considered, and the possibility of attempting specific treatments should be contemplated.

## Data Availability

No datasets were generated or analysed during the current study.

## Abbreviations

*CACNA1A*: Calcium voltage-gated channel subunit alpha1 A
Cav2.1: Calcium voltage-gated channel subunit alpha1 A
NGS: Next-generation sequencing
DNA: Deoxyribonucleic acid
CA: California
USA: United States of America
FASTQ: A file format used to store DNA sequence and corresponding quality information
SEQ: Sequence
CDS: Coding sequence
IGV: Integrative Genomics Viewer
ACMG: American College of Medical Genetics and Genomics
MRI: Magnetic resonance imaging
EEG: Electroencephalography
VUS: Variant of uncertain significance
FHM: Familial hemiplegic migraine
EA2: Episodic ataxia type 2
CBD: Cannabidiol
GABA: Gamma-amino butyric acid
ID: Intellectual disability
ASD: Autism spectrum disorder
ADHD: Attention-deficit/hyperactivity disorder
BPT: Benign paroxysmal torticollis
BPV: Benign paroxysmal vertigo
BTU: Benign paroxysmal tonic upgaze
SCA6: Spinocerebellar ataxia type 6

## Code availability

N/A.
